# Development of biochar-based magnetic adsorbent for enhanced Pb(II) ions removal

**DOI:** 10.1038/s41598-026-67881-4

**Published:** 2026-09-03

**Authors:** Soha M. Abd El Wahab, Kareem Elsayed, Hanaa A. Zein El Abdeen, Shimaa M. Ali

**Affiliations:** 1 Physics Department, Faculty of Science, Cairo University, Giza, 12613 Egypt; 2 Soil, Water and Environmental Research Institute, Agriculture Research Center (ARC), Giza, 12613 Egypt; 3 Chemistry Department, Faculty of Science, Cairo University, Giza, 12613 Egypt

**Keywords:** Biomass, Biosorbents, Magnetic properties, XRD, Wastewater treatment, Chemistry, Environmental sciences, Materials science

## Abstract

Waster decontamination by Pb(II) is a critical issue that threatens the environmental and human health. Ferrite nanoparticles NCCF, Ni₀.₆Co₀.₁Cu₀.₃Fe₂O₄, loaded on biochar (BC) in different ratios, 0.2 and 0.5 g, are prepared using the hydrothermal technique. X-ray diffraction (XRD), FT-IR spectroscopy, surface area measurements and vibrating sample magnetometer (VSM) are carried out to study the structural, surface characterizations and magnetic properties. XRD pattern identify single phase spinel formation. Biochar has negligible effect on the ferrite crystal structure but reduces lattice strain and promotes structural ordering, mainly through defect formation rather than structural modification. FT-IR indicates that the cubic spinel type has been efficiently deposited on the biochar surface. BET analysis showed that the surface area and pore volume of ferrite are increased at lower biochar loading. VSM analysis revealed that the incorporation of biochar into the ferrite structure decreases the saturation magnetization while preserving moderate coercivity. The prepared samples are used as biosorbents for Pb(II) ions removal from aqueous solutions. Studying the effect of the solution pH revealed that the addition of biochar into the ferrite structure improves adsorption effectiveness under acidic conditions. The adsorption follows the pseudo second order kinetics, implying a chemisorption mechanism through a complexation of surface functional groups. The Freundlich model best explained the adsorption behavior, indicating heterogeneous adsorption sites. The incorporation of biochar significantly enhanced the adsorption capacity, reaching a maximum adsorption capacity of 398.4 mg g⁻¹ for NCCF/BC-0.2, which is substantially higher than those of bare NCCF and BC. In addition, the prepared composites demonstrated good regeneration ability and maintained high Pb(II) removal efficiency in the presence of competing metal ions.

## Introduction

Contamination of water systems by heavy metals continues to be a key issue in environmental pollution owing to their non-degradable and accumulation properties. Among these heavy metals, one that is highly toxic and poses serious harm to the environment is Lead (Pb), which has widespread applications in various sectors including batteries, metal alloys, pigments, mining, and electroplating industries^1,2^. Pb(II) is associated with high bioaccumulation property and causes nervous system problems, kidney problems, cardiovascular issues, and blood diseases. Additionally, it has been established that long-term exposure to Pb, even in small amounts, leads to irreparable damage to the mental functioning in children and increased risk of heart disease in adults^3–8^. The chemical behavior of Pb(II) in water mainly depends on the solution pH and the redox potential (Eh). According to the Pb–H₂O Eh–pH (Pourbaix) diagram^9^, Pb²⁺ is the predominant dissolved species under acidic and near-neutral conditions, while lead hydroxides and other insoluble forms of Pb are favored with high pH values. Hence, pH has an important influence on whether Pb(II) is solubilized in solution or precipitated and thus affects the adsorption process^9,10^. An insight into the aqueous speciation of Pb(II) is vital in order to optimize adsorption conditions for Pb removal from solutions.

The treatment of industrial wastewater becomes an environmental issue of increasing significance as a consequence of the rapid development of industrial activities. Methods evaluated for removing these contaminants include chemical precipitation^11^, electrochemical processes^12^, biological treatments^13^, and other conventional physicochemical methods^14,15^. These methods are effective in contaminant removal; nevertheless, visit complicated operations, with high operational costs and secondary pollution problems^16^.

Among the various treatment technologies, adsorption has received particular attention because of its economic vibrancy, environmental friendliness, easier operation and higher efficiency in pollutant removal^14,17,18^.

Biochar is a porous carbonaceous material, typically containing more than 50 wt% elemental carbon, formed by the thermochemical conversion of biomass at temperatures between 300 and 700 °C. in an oxygen-deficient condition. Wood chips, crop wastes, animal dung, carcasses, sludge, and leaves are commonly used as raw material for the production of biochar^19^. Biochar has recently gained considerable attention for environmental remediation and bioenergy applications because of its potential to sequester carbon from the atmosphere and enhance soil fertility^20^. It has many outstanding properties such as a highly porous structure, high specific surface area, various surface functional groups and good chemical stability^21^. Recent studies have highlighted innovations in biochar with regard to increase of adsorption and desorption performance through the generation of microporous structures, an increase in quantity of surface functional groups and use of different modification approaches^22,23^. Among these modification methods, the preparation of composite materials by loading metal oxides onto wood biochar is an effective method to improve adsorption performance^24^.

Spinel ferrites are a type of magnetic material composed of ferric ions and have the general chemical formula M²⁺Fe₂³⁺O₄, where M represents a divalent metal ion such as Mg^2+^, Ni^2+^, Fe^2+^, Cu^2+^, or Co^2+^. Mixed ferrites can be prepared by substituting two or M^2+^ ions, dissimilar to each other in order to modulate their physical properties for specific applications. These materials are characterized by their high DC resistivity as well as excellent magnetic behavior^25^. Magnetic spinel ferrites have attracted significant interest for water treatment applications because of their excellent thermal and chemical stability, along with their unique structural and magnetic properties^26,27^. Morphologically, Spinel ferrites nanoparticles have a high surface area-to-volume ratio and porous structure allowing them to interface more with heavy metal ions in water, increasing the efficiency of their adsorption^28^. One major strength of iron spinel ferrites is their surface charge based on the point of zero charge (PZC), whose value influences the surface charge as follows: when the pH of the solution is lower than PZC, then the surface will acquire a positive charge, hence promoting the adsorption of anions like As(V) and Cr(VI). However, when the pH is higher than PZC, the surface will become negatively charged, hence allowing the adsorption of cations like Pb²⁺, Cd²⁺, and Cu²⁺^29,30^. This pH-responsive behavior makes Fe-based spinel ferrites versatile adsorbents for the removal of both anionic and cationic pollutants from wastewater. One of the most appealing features of these ferrites is their strong magnetic behavior, which allows for easy separation of the nanoparticles from treated water using an external magnetic field. Another major benefit is that these magnetic adsorbents can be applied several times without losing efficiency in the adsorption process, making possible a sustainable and low-cost process. Also, Spinel ferrites is an environmentally friendly option for water purification because it does not have harmful by-products associated to the treatment^31,32^.

In this work, biochar (BC)-based ferrite magnetic nanocomposites with a chemical formula Ni_0.6_Co_0.1_Cu_0.3_Fe_2_O_4_/BC are synthesized using the hydrothermal method with different biochar ratios of 0.2 and 0.5. The characterizations of the structural and surface properties are conducted via X-ray diffraction (XRD) and Fourier transform infrared (FTIR), and the specific surface area, BET analysis: Brunauer–Emmett–Teller. Vibrating sample magnetometry (VSM) is used to determine the magnetic properties of the synthesized nanocomposites. The obtained biochar–ferrite composites are applied as adsorbent materials for the removal of Pb(II) ions from aqueous solutions. The removal process of Pb(II) ions is systematically examined throughout several parameters that are as follows: solution pH, contact time, and initial concentration of the Pb(II) ions. In addition, the regeneration and reusability of adsorbents are evaluated, while the interference effect of potential interfering ions on adsorption performance is also investigated.

## Results and discussion

### Structural characterization

#### X-rays diffraction (XRD)

**Fig. 1:**
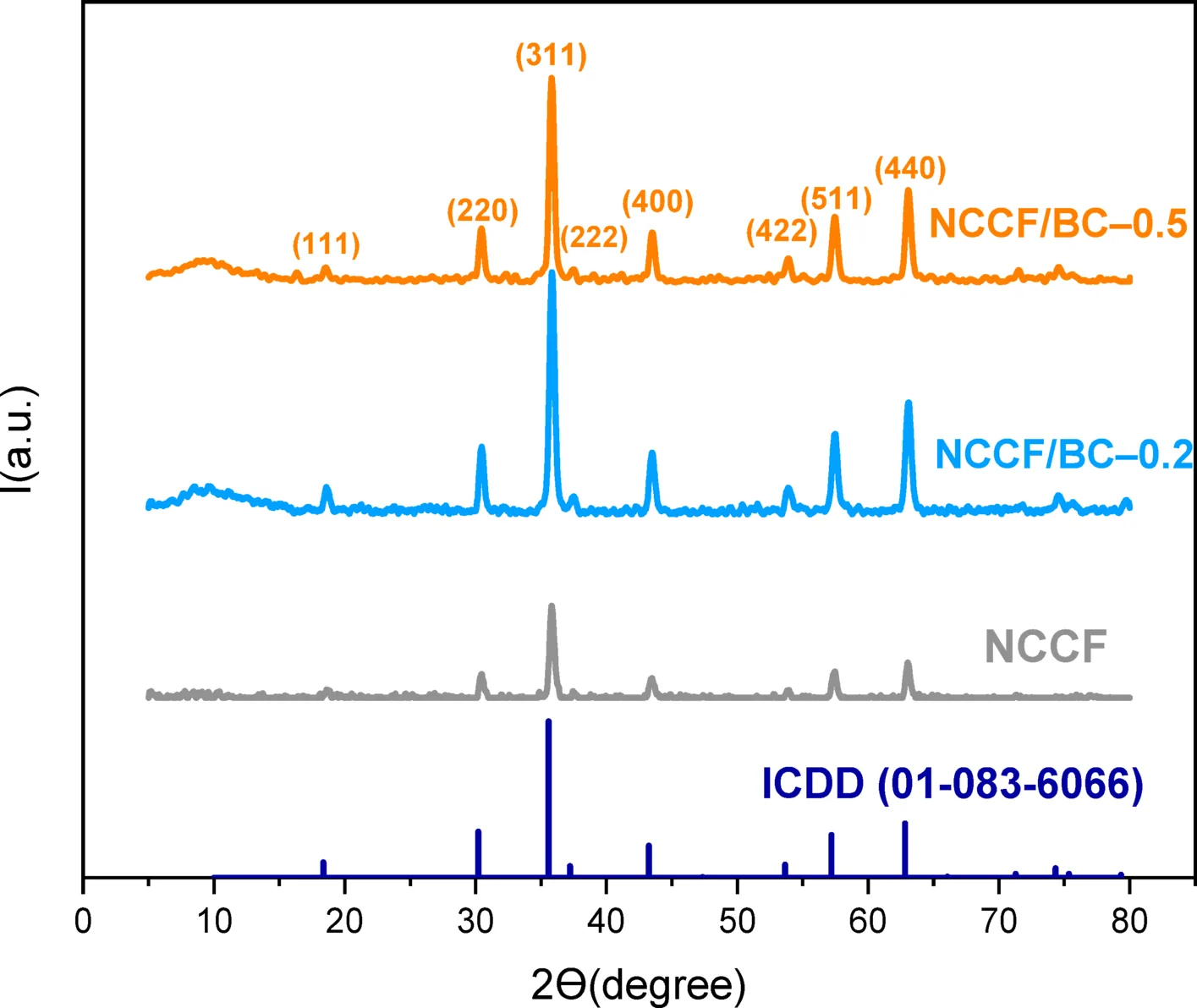
X-ray diffraction patterns for (NCCF) and NCCF/BC composites.

XRD investigation shows the crystal structure and phase purity of the samples under investigation. Figure 1 presents XRD patterns with all indexed peaks of (NCCF) and NCCF/BC composites corresponding to ICDD (01-083-6066) of Ni_0.5_Co_0.5_Fe_2_O_4_. The compounds had maximum peaks intensity at (311) and strong diffraction peaks at (111), (220), (222), (422), (511), and (440) which matched with the reflections of the face-centered cubic (FCC) single phase spinel structure with the Fd-3 m space group. It implies that the prepared samples are in a single phase free of contaminants.

The peak positions are matched to ICDD Card (01-083-6066) of Ni_0.5_Co_0.5_Fe_2_O_4_^33^. The NCCF/BC composites pattern resembles a hybrid of the (NCCF) and biochar patterns, with humping between 10° and 20°, which is attributed to the amorphous structure of the biochar^34^. The diffraction peaks of NCCF/BC composites are identical to those of the as-prepared NCCF, showing that the adding biochar does not promote phase transition. Table 1 shows XRD structure parameters such as average crystallite size (D), lattice parameter (a), volume of unit cell (V), dislocation density (δ), and The Lattice strain (ɛ) for produced samples.

The interplanar spacing (d) was calculated according to Bragg’s equation^35^:

1 $$\:n\lambda\:=2d\mathrm{si}\mathrm{n} \theta $$

The lattice parameter of the cubic spinel structure was evaluated using the relation^35^:

2 $$\:a=d\sqrt{{h}^{2}+{k}^{2}{+l}^{2}}$$

where (hkl) represent the Miller indices of the corresponding diffraction plane.

The average crystallite size (D) was estimated from the most intense diffraction peak using the Debye–Scherrer equation^36^:

3 $$\:\mathrm{D}=\frac{\mathrm{k}}{{{\upbeta\:}}_{\mathrm{h}\mathrm{k}\mathrm{l}}\mathrm{cos}}$$

where k is the shape factor (0.94 for cubic symmetry), λ is the wavelength of the Cu Kα radiation (0.15406 nm), β is the full width at half maximum (FWHM) of the diffraction peak expressed in radians, and θ is the Bragg diffraction angle.

The dislocation density (δ), which indicates the amount of crystallographic imperfections within the material, was determined using^37^:

4 $$\:{\updelta\:}=\frac{1}{{D}^{2}}$$

Furthermore, the lattice strain (ε) arising from structural distortions such as dislocations and stacking faults was calculated using^38^:

5 $$\:{\upvarepsilon\:}=\frac{{\upbeta\:}}{4\mathrm{tan}}$$

**Table 1:** The XRD structure parameters: average crystallite size (D), lattice parameter (a), volume of unit cell (V), dislocation density (δ), and lattice strain for NCCF and NCCF/BC composites.

Sample	D (nm)	a (A°)	V (A° ^3^)	δ x 10^− 3^ (nm^− 2^)	ɛ x 10^− 3^
NCCF	19.42	8.727	664.65	2.650	6.077
NCCF/BC–0.2	19.14	8.726	664.43	2.727	5.201
NCCF/BC–0.5	19.55	8.731	665.67	2.613	5.084

Through structural analysis it is confirmed that biochar does not have major impact either on the crystallographic structure of NCCF ferrite as NCCF maintains cubic spinel phase with minor variations in terms of crystallite size (~ 19 nm), lattice parameter and unit cell volume. On the other hand, as the biochar concentration increases, A significant decrease in lattice strain suggests reduced internal tensions and better structural ordering. The small variation in dislocation density indicates that biochar mostly forms defects which alter the properties from their normal behavior, rather than modifying crystal structure.

Crystallite size and microstrain both contribute to XRD peak broadening. To investigate the peak broadening in the XRD pattern, the crystallite size and microstrain are measured using two well-known models: the Williamson-Hall (W-H) plot and the Size-Strain Plot (SSP) method. The W-H method divides peak broadening into size and strain contributions using the following equation^39^:

6 $$ \beta {\mathrm{cos}}\theta {\text{ }} = {\text{ }}\left( {{\mathrm{k}}\lambda /{\mathrm{D}}} \right){\text{ }} + {\text{ 4}}\varepsilon {\text{ sin}}\theta $$

In contrast, the SSP model assumes Gaussian strain broadening and Lorentzian size broadening, which are expressed as:

7 $$ \left( {{\mathrm{d}}_{{{\mathrm{hkl}}}} \beta _{{{\mathrm{hkl}}}} {\mathrm{cos}}\theta } \right)^{{\mathrm{2}}} = {\text{ }}\left( {{\mathrm{k}}\lambda /{\mathrm{D}}} \right){\mathrm{.d}}_{{{\mathrm{hkl}}}} ^{{\mathrm{2}}} {\text{ }}\beta _{{{\mathrm{hkl}}}} {\mathrm{cos}}\theta {\text{ }} + {\text{ }}\varepsilon ^{2} /4 $$

**Fig. 2:**
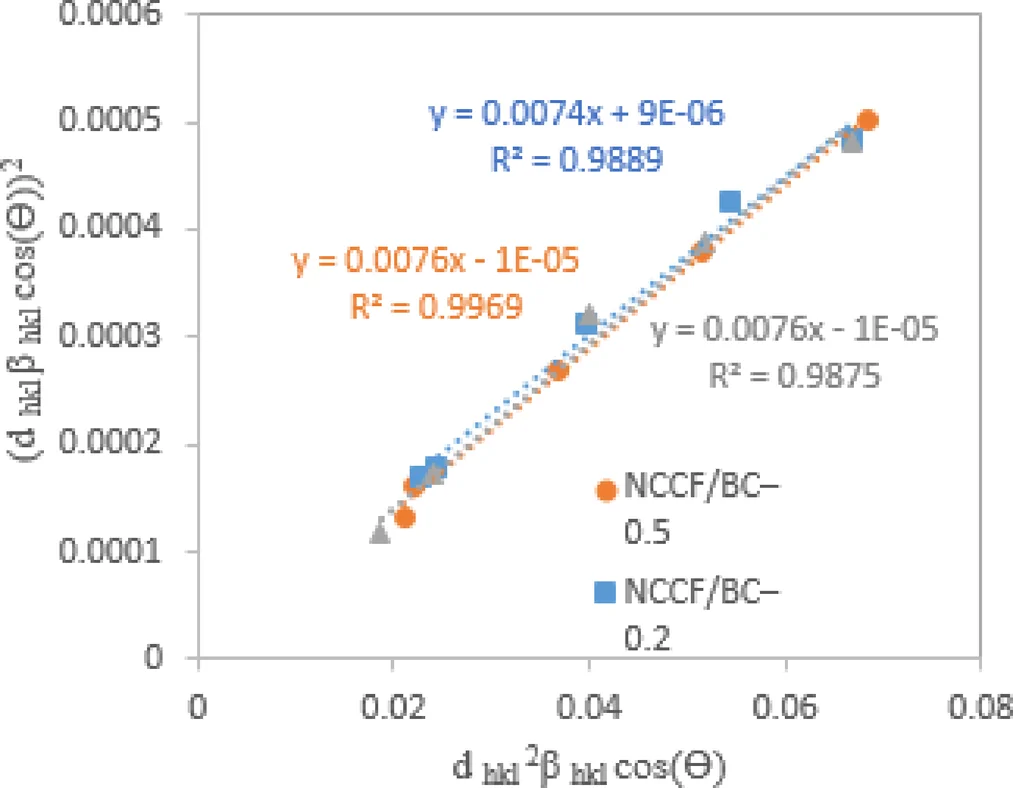
Size-strain plot (SSP) plot for NCCF and NCCF/BC composites.

**Table 2:** Crystallite size and microstrain of NCCF and NCCF/BC composites by the size-strain plot (SSP) method.

Sample		NCCF	NCCF/BC–0.2	NCCF/BC–0.5
Size-Strain Plot	Crystallite size (nm)	19.05	19.56	27.32
	Strain	6.32 × 10^− 3^	5.9 × 10^− 3^	7.6 × 10^− 3^

From Table 2, SSP results showed larger crystallite sizes (19.05–27.32 nm) and more pronounced positive strain (5.9–7.6 × 10⁻³). The SSP analysis demonstrates high accuracy compared to the Williamson–Hall method, with R² values nearly equal to one, as shown in Fig. 2. However, the linearity observed from the Williamson-Hall plots was not good enough to allow extraction of any quantifiable crystallite size or micro-strain data. Williamson-Hall technique was kept only for the purpose of comparison with the SSP technique. The W-H analysis showed poor linearity implying that the required assumptions are not completely met in case of synthesized nanoparticles. The W-H analysis requires the assumption that the broadening of the peaks is attributed to the combined effects of the crystallite size and isotropic lattice strain^40,41^. On the other hand, spinel ferrites generally show anisotropic strain because of the cationic rearrangement, lattice imperfections, and structure distortion violating the required assumption of the W-H analysis. On the other hand, the Size-Strain Plot (SSP) method offered a better linear relationship since it gives greater weight to low-angle reflections, thus resulting in reliable estimation of crystallite size and strain in ferrite nanoparticles^41^.

The SSP approach is generally regarded as more accurate when the lattice strain is relatively low, as it reduces the overestimation of size broadening and provides a better separation between size and strain contributions^39^. Therefore, the measured crystallite size and microstrain values obtained in the present study are based on the SSP only. The increase in crystallite size upon biochar incorporation, particularly at higher loading (0.5 g), suggests that biochar may facilitate crystal growth and partially relieve internal stresses.

#### Fourier transform infrared (FT-IR)

**Fig. 3:**
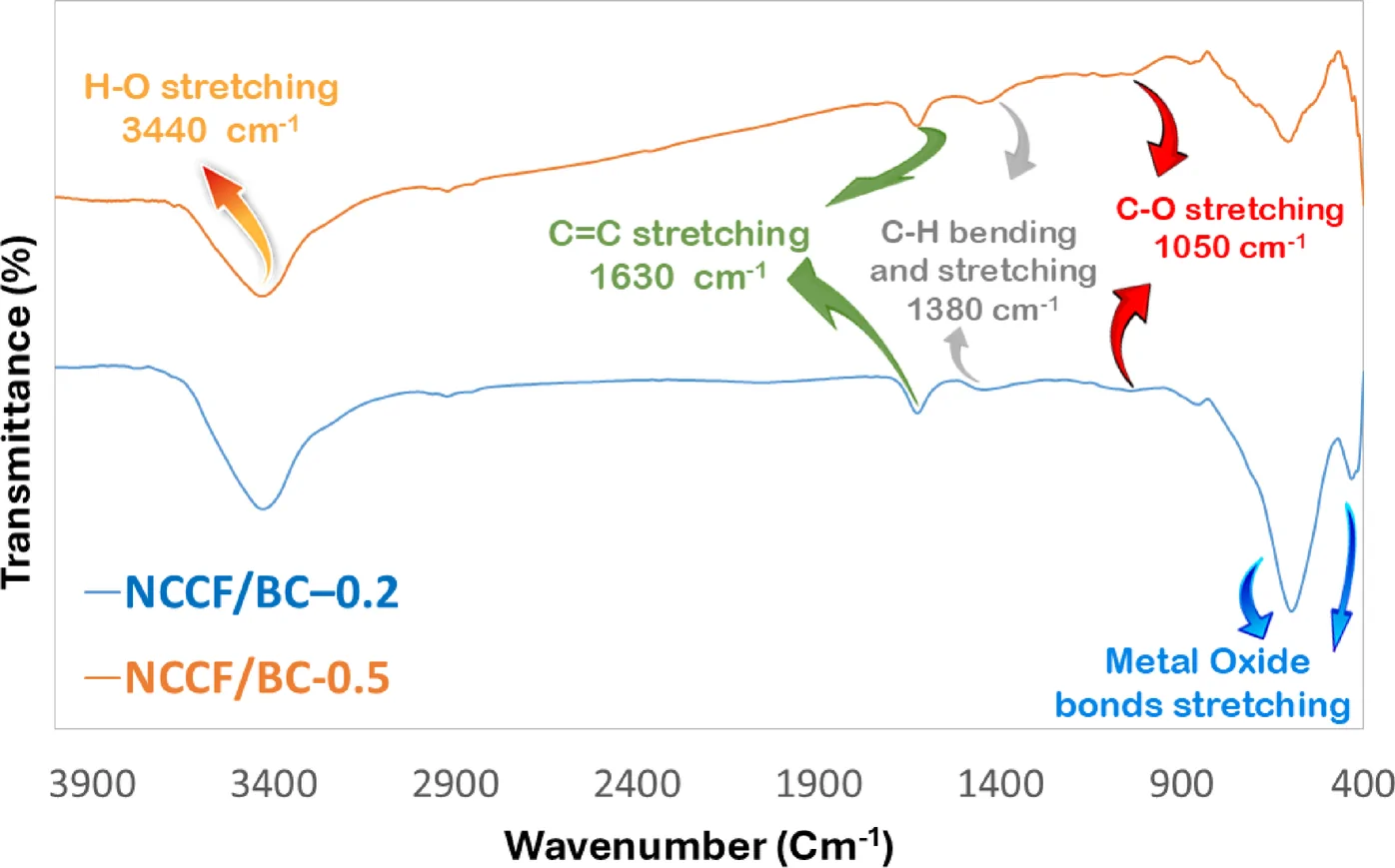
FT-IR of NCCF/BC composites.

FT-IR analysis further supported the spinel phase formation by revealing the presence of prominent absorption bands in the low-frequency region, specifically around **600 cm**^**-1**^ and **450 cm**^**-1**^. The bands observed in this study correspond to the intrinsic stretching vibrations of metal-oxygen bonds in the tetrahedral and octahedral complexes, respectively^42^, shown in Fig. 3. This confirms that the cubic spinel-type NCCF (Ni₀.₆Co₀.₁Cu₀.₃Fe₂O₄) had been effectively deposited on the biochar surface^43^.

Furthermore, the FT-IR measurements provided confirmation of the functional groups found on the surface of the composite materials. The stretching vibration of O-H (from hydroxyl groups or adsorbed moisture) was identified as the cause of the broad peak observed at around **3440 cm**^**-1**^. The absorption band at approximately **1630 cm**^**-1**^ corresponds to the stretching vibrations of C = C bonds in the aromatic rings of the biochar framework^44^. Additionally, the peaks near **1380 cm**^**-1**^ and **1050 cm**^**-1**^ are attributed to C-H bending and the stretching vibrations of oxygen-containing functional groups (such as carboxylic or alkoxy C-O groups) on the biochar, respectively^45^. The coexistence of these distinctive biochar peaks alongside the characteristic metal-oxygen bands provides strong evidence of the successful combination of NCCF with BC.

### Surface characterizations

#### Brunauer-Emmett-Teller (BET) surface area measurements

**Fig. 4:**
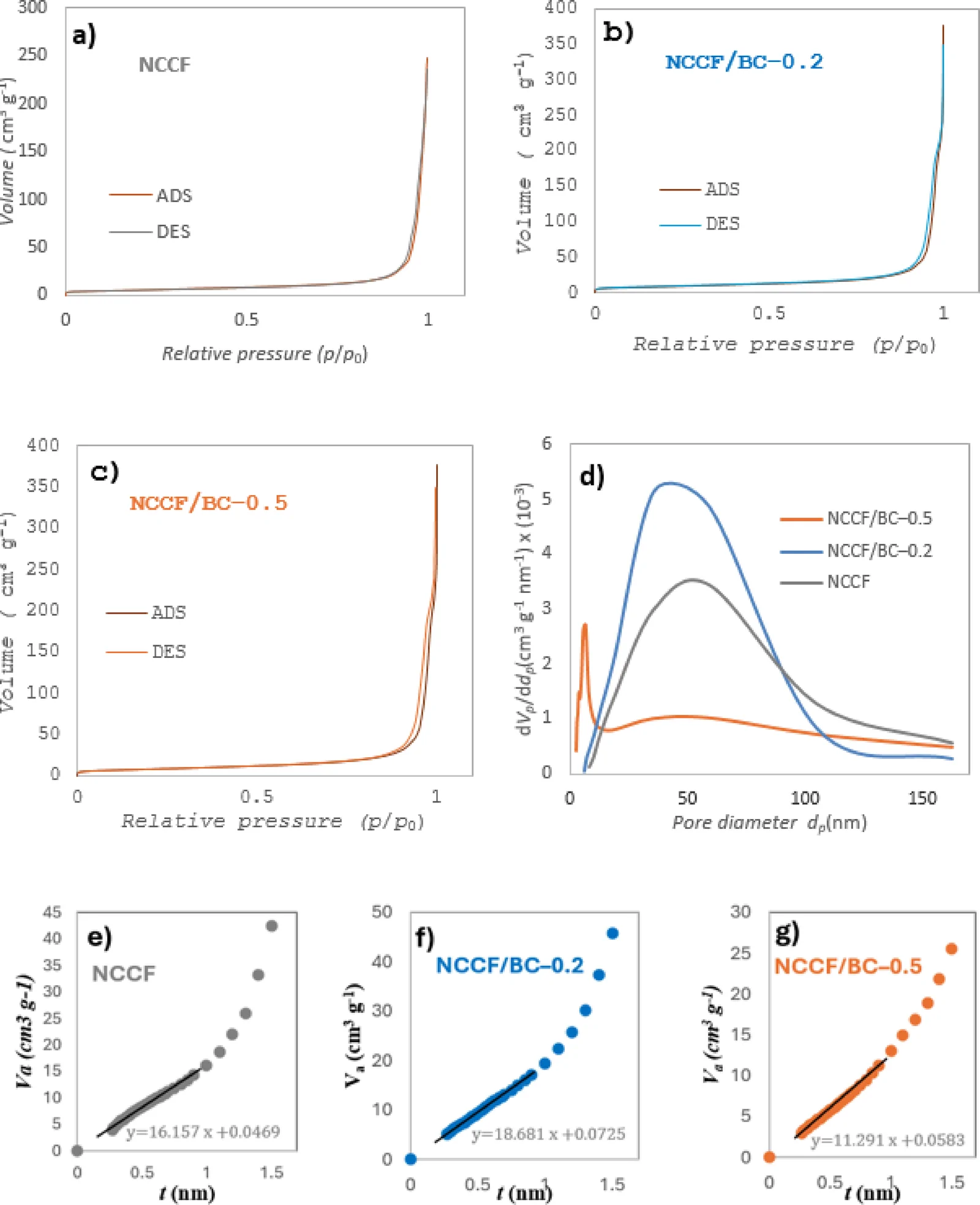
(**a**–**c**) Adsorption/desorption isotherm, (**d**) pore size distribution using BJH methods & (**e**–**g**) t-plot method for (NCCF) and NCCF/BC composites.

The textural properties of the **NCCF and NCCF/BC composites** were examined by nitrogen adsorption–desorption measurements at 77 K, following degassing at 200 °C for 8 h. As illustrated in Fig. 4a–c, the adsorption isotherms of all samples can be classified as *Type IV* according to the IUPAC classification^46^, exhibiting a single-point saturation plateau and an H3-type hysteresis loop^47^.

This behavior is characteristic of mesoporous materials composed of non-rigidly aggregated particles, leading to the formation of slit-shaped pores^48^. The specific surface area, total pore volume, and average pore diameter were determined using the BET method^49^, and the corresponding values are summarized in Table 3.

BET surface area and porosity analyses revealed significant changes upon biochar loading. The obtained NCCF that featured a mesoporous structure through interparticle voids of ferrite nanoparticles, had a moderate specific surface area of 20.67 m² g⁻¹ and total pore volume of 0.26 cm³ g⁻¹. On addition of 0.2 g biochar (NCCF/BC–0. 2), the surface area and pore volume of BET trends were substantially enhanced to 29.28 m² g⁻¹ and 0.33 cm³ g⁻¹, respectively. This enhancement can be attributed to the highly porous nature of biochar and its role in preventing ferrite nanoparticle agglomeration, which generates additional pores. However, further increasing the biochar content to 0.5 g (NCCF/BC–0.5) resulted in a decrease in surface area (17.89 m² g⁻¹) and pore volume (0.13 cm³ g⁻¹), which is likely due to high amount of biochar allows iron oxides to enter and block some of the biochar pores^50^. From the t-plot method, NCCF/BC-0.2 was found to have the greatest external surface area (18.68 m² g⁻¹) and micropore volume (0.0725 cm³ g⁻¹), suggesting that there was greater pore accessibility and dispersal of ferrite nanoparticles after the introduction of biochar. An increase in biochar content to 0.5 g resulted in lower values of both external surface area and micropore volume, which could be attributed to the blockage of some pores and aggregation of particles^50^. These results, together with BJH analysis, confirmed that all samples possess mesoporous characteristics, making NCCF/BC–0.2 the most favorable candidate for adsorption-based environmental applications.

**Table 3:** The total pore volume, BET average pore size, BET surface area, external surface area micropore volume of (NCCF) and NCCF/BC composites.

Sample	Total pore volume (cm^3^/g)	BET average pore size (nm)	BET surface area (m^2^/g)	External surface area (m²/g)	Micropore volume (cm³/g)
NCCF	0.2634	50.960	20.676	16.157	0.0469
NCCF/BC–0.2	0.3251	44.409	29.282	18.681	0.0725
NCCF/BC–0.5	0.1280	28.627	17.888	11.291	0.0583

### Magnetic properties

**Fig. 5:**
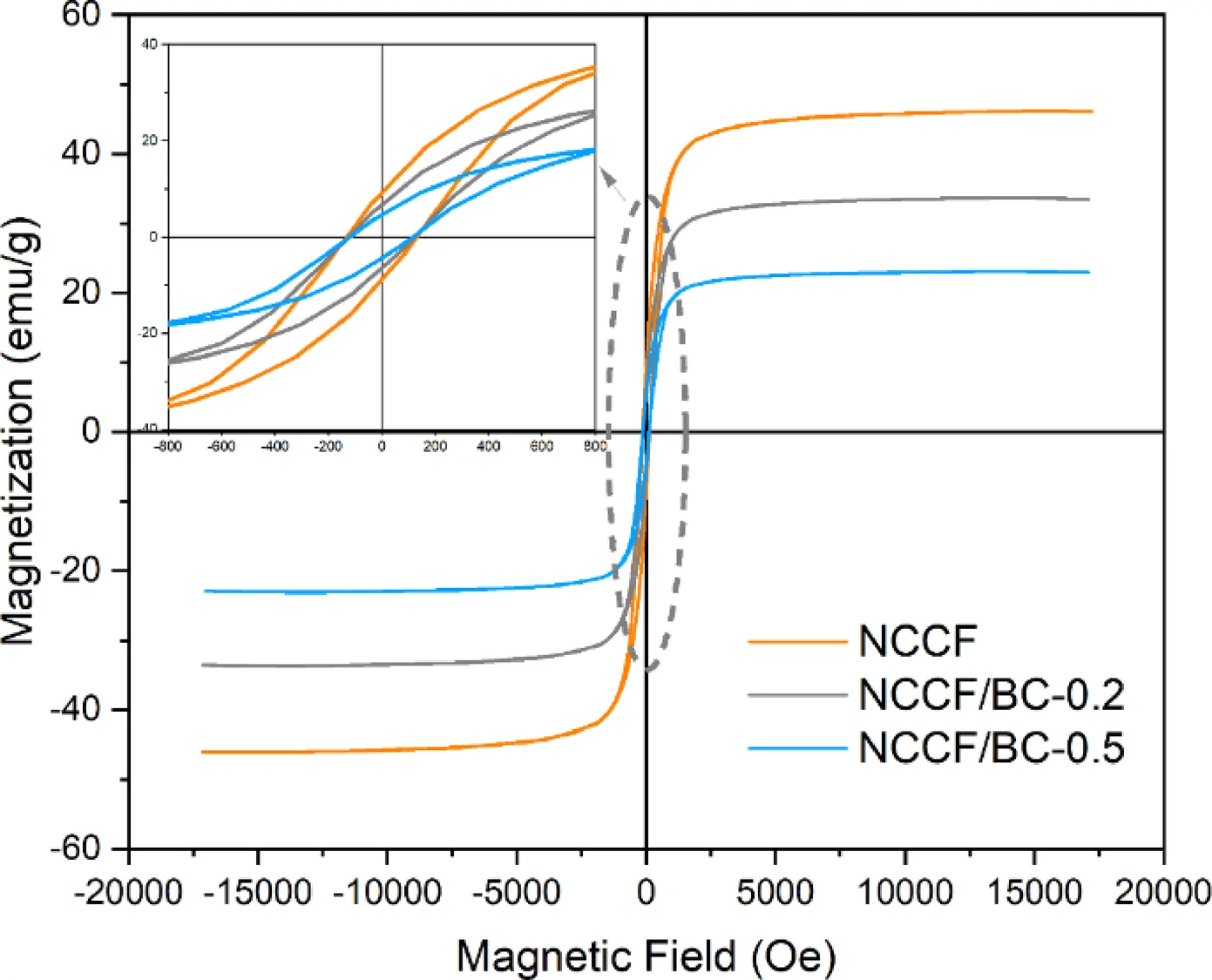
Magnetic hysteresis curves of (NCCF) and NCCF/BC composites at 300 K.

The magnetic properties were investigated using vibrating sample magnetometer (VSM) at room temperature. Figure 5 displays the magnetic hysteresis loops of (NCCF) and NCCF/BC composites at 300 K, and the derived magnetic parameters are summarized in Table 4. The saturation magnetization (Ms), remanent magnetization (Mr) and coercivity (Hc) of the pure NCCF sample were 46.15 emu g⁻¹, 8.94 emu g⁻¹ and 131.14 Oe respectively, suggesting that it exhibited typical ferrimagnetic characteristics for a spinel ferrite^51^. by loading of biochar (BC), both M_s_ and M_r_ decreased with increasing BC content. The M_s_ value decreased to 33.48 emu g⁻¹ for NCCF/BC–0.2 and further to 23.02 emu g⁻¹ for NCCF/BC–0.5, while M_r_ decreased from 8.94 to 6.63 and 4.55 emu g⁻¹, respectively. Such decrease is due to reduction of the magnetic phase by the non-magnetic biochar matrix, which weakens metal ion interactions through superexchange at either tetrahedral (A) or octahedral (B) sites in the spinel structure for a reduced net magnetic moment.

The remanence ratio (R = Mr/Ms) was approximately constant across all samples (~ 0.19–0.20), indicating that the magnetic domain configuration is largely independent of the addition of BC. In contrast, the coercivity showed a slight decrease from 131.14 Oe for pure NCCF to 123.34 Oe and 117.41 Oe for NCCF/BC–0.2 and NCCF/BC–0.5, respectively. This observed reduction is ascribed to the reduced interparticle magnetic interactions resulting from the presence of carbon matrix, which partially separates the ferrite nanoparticles. Furthermore, surface effects and possible slight differences in particle size may affect magnetic anisotropy. Comparative drops in coercivity and magnetization have been noted for ferrite–biochar composites, attributed to the non-magnetic carbon phase which acts on both magnetic coupling as well as structural properties^52^.

**Table 4:** Saturation magnetization (Ms), remanent magnetization (M_r_), squareness ratio (R), and coercivity (Hc) for (NCCF) and NCCF/BC composites.

Sample	M_s_ (emu/g)	M_*r*_ (emu/g)	*R*	H_c_ (Oe)
NCCF	46.15	8.94	0.193	131.14
NCCF/BC–0.2	33.48	6.63	0.198	123.34
NCCF/BC–0.5	23.02	4.55	0.197	117.41

Overall, the results indicate that incorporating biochar into the NCCF structure effectively changes the magnetic properties by reducing saturation magnetization while maintaining moderate coercivity. Despite the reduction in magnetization, the composites still have sufficient magnetic response, which is advantageous for applications requiring magnetic separation, such as wastewater treatment and catalytic processes.

### Application of biochar-based magnetic adsorbents for the Pb(II) ions removal

#### Effect of the solution pH

**Fig. 6:**
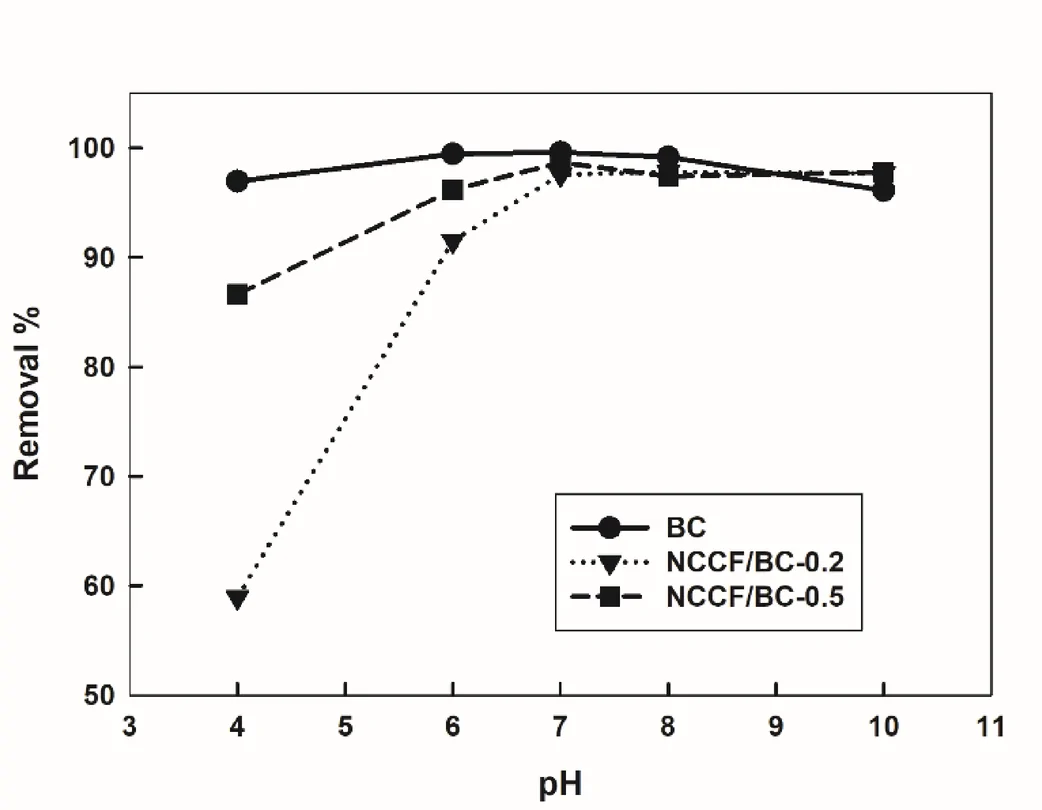
Variation of the removal % of Pb(II) ions by BC, and NCCF/BC composites with the pH, the initial Pb(II) ions concentration = 30 ppm, shaking time = 24 h, at room temperature.

The solution pH has a vital role in determining the adsorption mechanism and the removal efficiency. Several mechanisms have been reported for the removal of Pb(II) ions by biochar-based adsorbents such as; physical adsorption, surface complexation, electrostatic interactions, ion exchange, and minerals precipitation^53^. Generally, at low pH values, a repulsion between the positively charged functionalized biochar and Pb (II) ions leads to a poor uptake. While, as the pH values increases > 3–4, the adsorption efficiency increases^54–56^. On the other hand, the point of zero charge, PZC, for NCCF, as reported in our previous work, is 5.2^48^. Figure 6 shows the variation of the removal efficiency of the bare biochar; BC, and the prepared magnetic nanocomposites; NCCF/BC-0.2 and NCCF/BC-0.5 with the solution pH. Based on the above findings, an expected trend is shown in Fig. 6, where the removal % is high at high pH values > 5. While, at low pH value of 4, the biochar maintains its excellent performance, PZC = 2–3^54^, the decrease in the removal % is related to the magnetic NCCF and it is more pronounced with increasing NCCF content in the composite, sample NCCF/BC-0.2.

#### Kinetic study

**Fig. 7:**
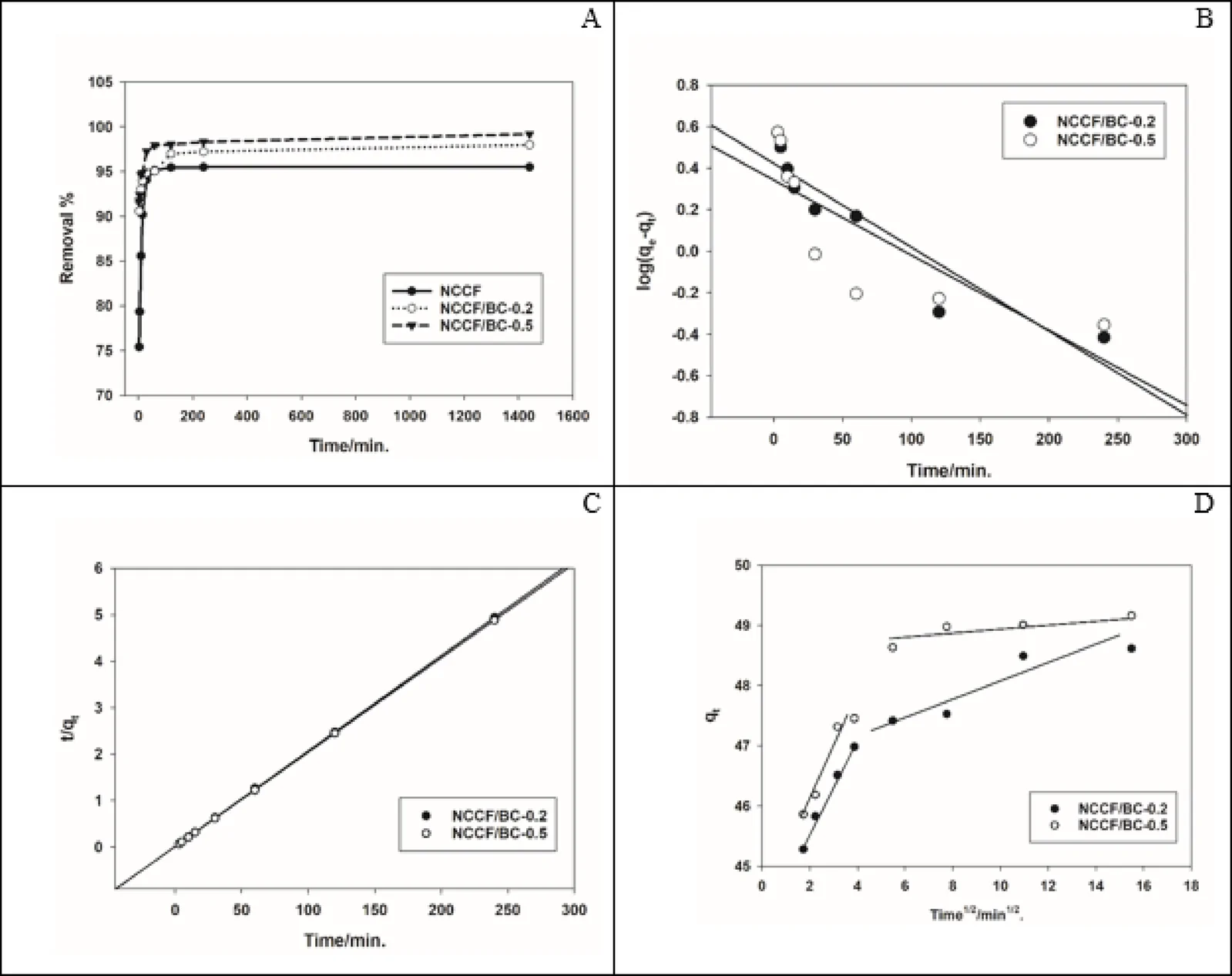
(**A**) Variation of the removal % of Pb(II) ions by NCCF, and NCCF/BC composites with the contact time, at pH = 6, the initial Pb(II) ions concentration = 100 ppm, at room temperature, (**B**) 1st, (**C**) 2nd order kinetic, and (**D**) the intra-particle diffusion model for NCCF/BC-0.2, and NCCF/BC-0.5.

The adsorption efficiency % of NCCF and prepared magnetic nanocomposites for the removal of Pb(II) ions, are calculated at different contact times. Figure 7A shows the variation of the removal % with the contact time. It is clear that introducing biochar in the nanocomposite facilitates the adsorption, especially at the early stages. The equilibrium is reached within 30 min. for NCCF while, a much faster uptake, within 10 min., is observed by introducing the biochar and it is more pronounced with increasing its amount.

Kinetic data are fitted to pseudo first, pseudo second kinetics, and the intra-particle diffusion models, as shown in Fig. 7B–D, respectively^57^.

8 $$\:\mathrm{log}\left({q}_{e}-{q}_{t}\right)=\mathrm{log}{q}_{e}-\frac{{k}_{1}}{2.303}\:t$$

9 $$\:\frac{t}{{q}_{t}}=\frac{1}{{k}_{2}{q}_{e}}+\:\frac{t}{{q}_{e}}$$

10 $$\:{q}_{t}={k}_{id}\sqrt{t}+\:C$$

where, q_t_ and q_e_ represent the amounts adsorbed at time = t and at equilibrium, respectively. k_1_ and k_2_ denote the pseudo first and second order rate constants of adsorption, respectively. k_id_ is the constant for intra-particle diffusion rate.

From calculated kinetic parameters listed in Table 5, The values of R^2^ are closer to unity, and the calculated adsorbed amounts at equilibrium, q_e_, are closer to the experimental values, in the case of the pseudo second order kinetics. Thus, it can be concluded that the adsorption of Pb(II) ions on magnetic NCCF, and prepared magnetic biochar-based composites follows the pseudo second order kinetics, suggesting a chemisorption mode through a complexation of surface functional groups^57,58^. By increasing the amount of the biochar, in the prepared composite, the kinetic of the adsorption is more facilitated, as indicated by the higher k_2_ values.

**Table 5:** Calculated kinetic data for the adsorption of Pb^2+^ ions by NCCF, and NCCF/BC composites samples for pseudo 1st, pseudo 2nd, and intraparticle diffusion models.

Adsorbent	q_e, exp_ mg.g^− 1^	pseudo 1st order kinetic model			pseudo 2nd order kinetic model		
		q_e, cal_ mg.g^− 1^	k_1_ min^− 1^	*R* ^2^	q_e, cal_ mg.g^− 1^	k_2_ mg.g^− 1^.min	*R* ^2^
NCCF^48^	42.81	6.61	0.03	0.7801	43.46	0.54	0.9998
NCCF/BC-0.2	48.99	2.63	570.05	0.8664	47.62	1.75	0.9999
NCCF/BC-0.5	49.56	2.19	636.19	0.6576	49.26	2.64	0.9999

Intra-particle diffusion model							
Adsorbent		Surface adsorption			Pore diffusion		
		***k***_***surf***_ **mg.g**^**− 1**^.**min**^**½**^	***C***_***surf***_	***R*** ^***2***^	***k***_***id***_ **mg.g**^**− 1**^.**min**^**½**^	***C***_***id***_	***R*** ^***2***^
NCCF^48^		3.06	25.70	0.9784	0.12	41.48	0.6896
NCCF/BC-0.2		0.78	44.00	0.9908	0.13	46.69	0.8535
NCCF/BC-0.5		0.81	44.47	0.9409	0.05	48.49	0.7908

Two linear sections are observed in the intra-particle diffusion models for NCCF/BC-0.2, and NCCF/BC-0.5 samples, as shown in Fig. 7D, suggesting that the Pb(II) ions are first adsorbed at the composite surface then, it is diffused into the sorbent pores^57^. Higher intercept values and lower rate constants are observed for the second linear sections related to the pore diffusion; in the case of NCCF, therefore, the pore diffusion is limited with respect to the surface adsorption, and can be considered as the rate-determining step for the adsorption of Pb(II) ions on the magnetic NCCF^48^. It is worth mentioning that in the presence of the biochar, both surface adsorption and pore diffusion can be attributed to the rate-determining step, since the values of surface and diffusion intercepts are comparable. This is due to the increased porosity of biochar-based composites relative to the bare NCCF.

#### Adsorption isotherms

**Fig. 8:**
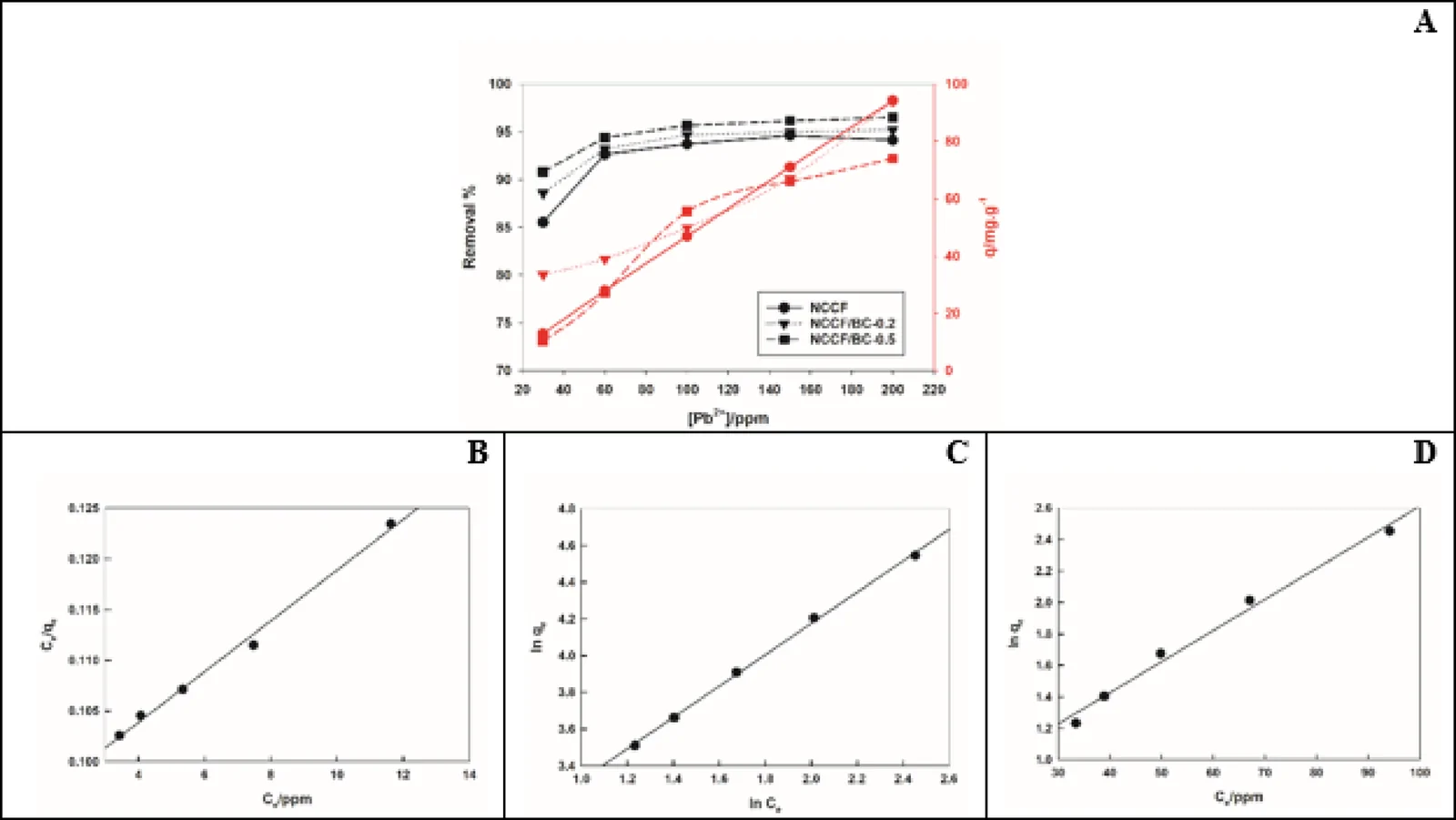
(**A**) Variation of the removal % and the adsorption capacity of Pb(II) ions by NCCF, and NCCF/BC composites with the initial metal ions concentration at pH = 6, shaking time = 2 h, at room temperature. (**B**) Langmuir, (**C**) Freundlich, and (**D**) Temkin adsorption isotherms for adsorption of Pb(II) ions on NCCF/BC-0.2.

The Variation of the removal % and the adsorption capacity of Pb(II) ions by NCCF, NCCF/BC-0.2, and NCCF/BC-0.5 with the initial metal ions concentration is shown in Fig. 8A. The removal % and the adsorption capacity increase by the increasing the initial Pb(II) ions concentration, and this is more pronounced with the increasing the biochar amount in the composite, reflecting the excellent adsorption performance of the prepared magnetic nanocomposites. The observed increase in removal efficiency with increasing of the initial metal ion concentration, within the evaluated concentration window, can be attributed to mass transfer kinetics and synergistic surface phenomena. High initial pollutant concentrations yield a concentration gradient that overcomes liquid film diffusion resistance, forcing pollutant species deeper into the micro/mesoporous network of the composite. Furthermore, for materials exhibiting cooperative adsorption behavior, the initial ion uptake enhances the affinity of the sorbent for the remaining ions via intermolecular interactions, which in turn outcompetes the background solvent molecules.

The adsorption data are fitted to Langmuir^59^, Freundlich^60^ and Temkin^61^ isotherms, as shown in Fig. 8B–D, respectively. According to the following Eqs.^62–64^, different isotherms parameters are calculated and listed in Table 6.

11 $$\:\frac{{C}_{e}}{{q}_{e}}=\frac{1}{{K}_{L}{q}_{m}}+\frac{{C}_{e}}{{q}_{m}}$$

12 $$\:\mathrm{ln}{q}_{e}=\mathrm{ln}{K}_{F}+\:\frac{1}{n}\mathrm{ln}{C}_{e}$$

13 $$\:{q}_{e}=\:\frac{RT}{b}\mathrm{ln}{K}_{T}+\:\frac{RT}{b}\mathrm{ln}{C}_{e}$$

where, *K*_*L*_, *K*_*F*_, and *K*_*T*_ are Langmuir, Freundlich, and Temkin constants, respectively. *q*_*m*_ is the maximum adsorption capacity (mg.g^− 1^). *n* and *b* are Freundlich and Temkin constants.

**Table 6:** Calculated adsorption isotherms parameters by fitting the adsorption data of Pb(II) ions by the prepared NCCF, and NCCF/BC composites samples to different isotherms.

Adsorbent	Langmuir			Freundlich			Temkin		
	K_L_ L.mg^− 1^	q_m_ mg.g^− 1^	*R* ^2^	K_F_ mg^1−(1/*n*)^.L^1/*n*^.g^− 1^	*n*	*R* ^2^	K_T_ L.mg^− 1^	b kJ.mol^− 1^	*R* ^2^
NCCF	0.120	**47.62**	0.9967	2.42	0.48	0.9991	0.93	6.10	0.9738
NCCF/BC-0.2	0.027	**398.40**	0.9938	11.82	1.18	0.9986	4.79	12.67	0.9880
NCCF/BC-0.5	0.041	**152.91**	0.9169	2.36	3.05	0.9991	11.02	1.03	0.9370

**Fig. 9:**
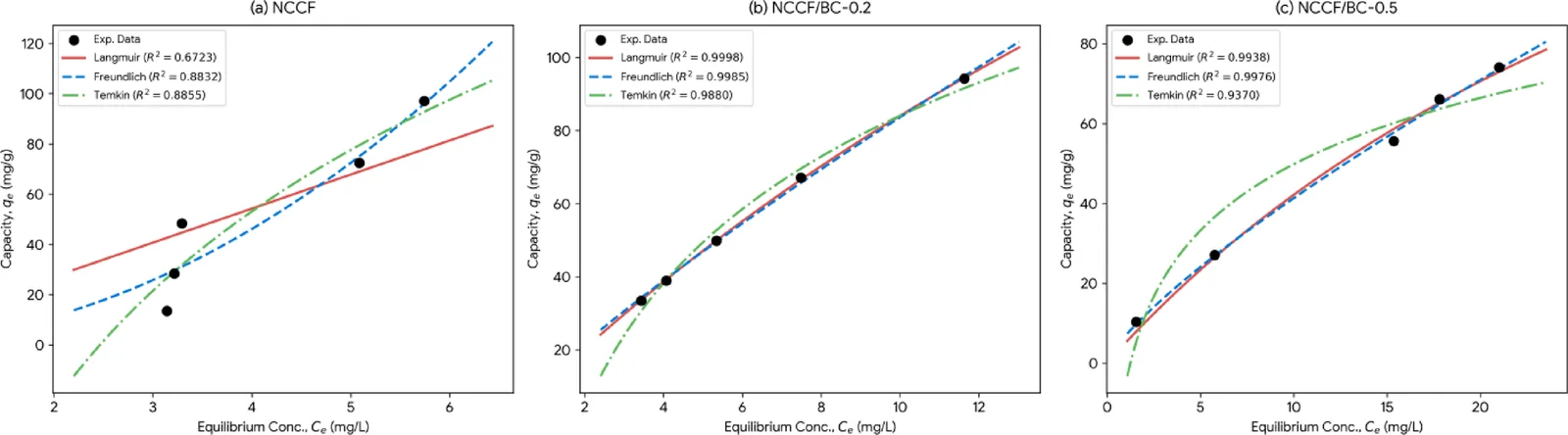
Non-linear fits for Langmuir, Freundlich, and Temkin models across (**a**) NCCF, (**b**) NCCF/BC-0.2, and (**c**) NCCF/BC-0.5.

Based on the correlation coefficient, R^2^, values, the adsorption of Pb(II) on NCCF, NCCF/BC-0.2, and NCCF/BC-0.5 fits best Freundlich isotherms, that is to say the adsorption occurs on non-energetically equivalent heterogenous sites. To further evaluate the adsorption isotherms, nonlinear regression was performed. As shown in Fig. 9; Table 7, samples NCCF and NCCF/BC-0.5, Freundlich can better describe the adsorption data (which agrees with the linear models). While for sample NCCF/BC-0.2, Langmuir is better. The calculated q_m_ values for NCCF/BC-0.2 and NCCF/BC-0.5, as presented in Table 7, were 388.28 and 217.49 mg.g⁻¹, respectively, indicating their high adsorption capacities. The same trend is observed which is the decrease in the q_m_ value as the biochar amount increases. For sample NCCF, the Langmuir isotherm failed to yield physically realistic parameters (q_m_=2.29 × 10^7^ mg/g, R^2^ = 0.6723) due to the absence of a saturation plateau within the measured concentration range.

**Table 7:** Non-linear parameters and goodness-of-fit metrics as indicated by regression coefficient, R^2^, root mean square error (RMSE) and Chi-square (χ^2^).

Sample	Model	Fitted Parameters	*R*²	RMSE	χ²
NCCF	Langmuir	q_m_ = 2.29 × 10⁷ mg/g, K_L_ = 5.91 × 10⁻⁷ L/mg	0.6723	17.199	30.5271
	**Freundlich**	K_F_ = 2.780 (mg/g)(L/mg)¹/ⁿ, *n* = 0.494 (1/*n* = 2.025)	**0.8832**	10.269	17.5041
	**Temkin**	A_T_ = 0.4054 L/mg, B = 109.760 J/mol	**0.8855**	10.167	16.0110
NCCF/BC-0.2	**Langmuir**	q_m_ = 388.275 mg/g, K_L_ = 0.0276 L/mg	**0.9998**	0.328	0.0093
	Freundlich	K_F_ = 12.223 (mg/g)(L/mg)¹/ⁿ, *n* = 1.197 (1/*n* = 0.835)	0.9985	0.852	0.0652
	Temkin	A_T_ = 0.5377 L/mg, B = 49.954 J/mol	0.9880	2.411	0.5819
NCCF/BC-0.5	Langmuir	q_m_ = 217.487 mg/g, K_L_ = 0.0240 L/mg	0.9938	1.894	0.9841
	**Freundlich**	K_F_ = 6.852 (mg/g)(L/mg)¹/ⁿ, *n* = 1.282 (1/*n* = 0.780)	**0.9976**	1.174	0.1521
	Temkin	A_T_ = 0.7975 L/mg, B = 23.992 J/mol	0.9370	6.055	8.4474

*The adsorption mechanism*, in the presence of biochar, is more complicated and favored by many factors. The presence of more oxygen-containing functional groups; OH and C-O groups, and also aromatic C = C groups, which provide π-electrons required for the surface complexation with the Pb(II) ions, as shown if FT-IR section, Fig. 3. Moreover, the enhanced porosity and the surface roughness^53–55,65^ resulted in effective surface area and promoted adsorption performance. The calculated maximum adsorption capacities for NCCF/BC-0.2 and NCCF/BC-0.5 are much higher than that for the bare NCCF, indicting the enhanced adsorption performance by introducing the biochar phase in the magnetic adsorbent. The adsorption capacity values are three-times and nine-times higher than that of the bare NCCF for biochar ratio of 0.5 and 0.2, respectively. Higher biochar ratio in NCCF/BC-0.5, still performs better than the bare magnetic ferrite NCCF, but the ratio required to be optimized because higher ratio reduce the porosity and the effective surface area. The maximum adsorption capacities reported for biochar materials, prepared by the pyrolysis of different agriculture wastes, range from 20 to 200 mg.g^− 1^^53^. In this study, introducing the magnetic ferrite to the biochar not only added a magnetic character to the adsorbent and thus a practical applicability, but also enhanced the adsorption performance, q_m_ for NCCF/BC-0.2 is 398.4 mg.g^− 1^.

To evaluate the practical competitiveness of NCCF/BC-0.2, q_m_ value, 398.4 mg.g^− 1^, is benchmarked against alternative commercial and state-of-the-art sorbents under comparable conditions. As summarized in Table 8, NCCF/BC-0.2 exhibits a capacity nearly double that of standard commercial activated carbons (150–250 mg/g) while operating efficiently at a low dosage ($\:1.0\mathrm{\:g/L}$) and neutral pH ($\:\mathrm{pH\:}6.0\mathrm{--}7.0$). Although pristine metal-organic frameworks (MOFs) can achieve higher raw capacities, their industrial adoption is hindered by high production costs and poor hydrothermal stability. In contrast, the biochar composite developed in this work offers a favorable balance of high uptake capacity, low cost, green synthesis, and operational robustness, confirming its genuine potential for industrial wastewater remediation.

**Table 8:** A literature benchmarking and comparative sorbent performance.

Sorbent material	qmax (mg/g)	Optimal pH	Dosage (g/L)	Key operational notes	Reference
NCCF/BC-0.2 composite	388.28	6.0–7.0	2.0	Monolayer chemisorption on biochar matrix; low energy input	This study
Commercial powdered AC (PAC)	150.00–250.00	5.0–7.0	1.0–2.0	Physical micropore filling; high energy footprint & cost	^66^
Durian shell activated carbon	180.00	7.0	2.5	Biomass waste carbon; lower mass transfer kinetics	^67^
Chitosan-biochar composite	310.00	6.0	1.5	Electrostatic attraction + hydrogen bonding	^68^
MIL-101(Cr) MOF	550.00	6.5	0.5	Ultra-high surface area; toxic synthetic route & low hydrolytic stability	^69^
Cu-BTC MOF / biochar hybrid	420.00	7.0	0.8	Enhanced open metal sites; metal leaching	^69^

#### Regeneration and interferences study

**Fig. 10:**
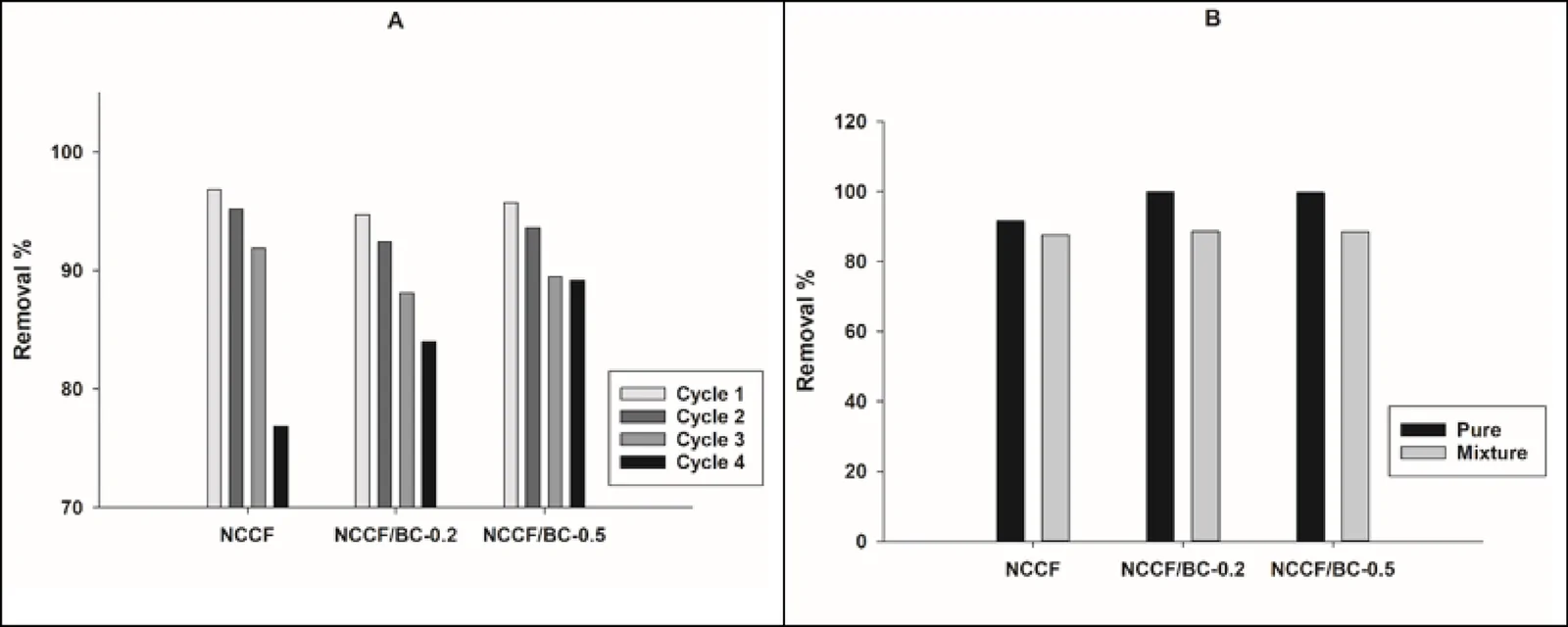
Variation of the removal % of NCCF, and NCCF/BC composites for the Pb(II) ion with (**A**) successive operation-regeneration cycles, and (**B**) in pure and mixed Pb(II) ion solutions.

The possibility of the adsorbent regeneration and the number of its operating adsorption cycles greatly influence the adsorbent applicability. In this work, the prepared NCCF/BC-0.2 sample is regenerated by being shaken in 1% HNO_3_ solution for 1 h. Figure 10A shows the variation of the removal % of NCCF, NCCF/BC-0.2, and NCCF/BC-0.5 samples for the Pb(II) ion with successive four operation-regeneration cycles. It can be shown that by increasing the biochar amount in the magnetic composite, the adsorbet maintains more its high adsorption performance especially after the third cycle. ICP analysis of the filtrates is performed after four successive regeneration/reuse of the presented samples. It was shown that no traces of Fe, Ni, Co, or Cu ions are present, indicating the stability of the proposed adsorbents.

The adsorption preformance of NCCF, NCCF/BC-0.2, and NCCF/BC-0.5 samples in the presence of interfering cations is examined by comparing the performance in individual Pb(II) ions solution and an equimolar mixture of Pb(II), Co(II), and Cd(II) ions, as shown in Fig. 10B. The biochar-based adsorbents peform a higher performance in pure Pb(II) ions solution, and exhibit a good removal efficiency for the Pb(II) ions removal in the presence of interfering cations.

## Method

### Chemicals

High purity ferric nitrate (> 98%), nickel (II) nitrate (> 97%), copper(II) nitrate (< 97%), cobalt(II) nitrate (< 97% (,lead nitrate (99%), nitric acid (69%), potassium hydroxide (< 96%) in pellets form were all obtained from Sigma–Aldrich.

### Synthesis of biochar

**Scheme 1:**
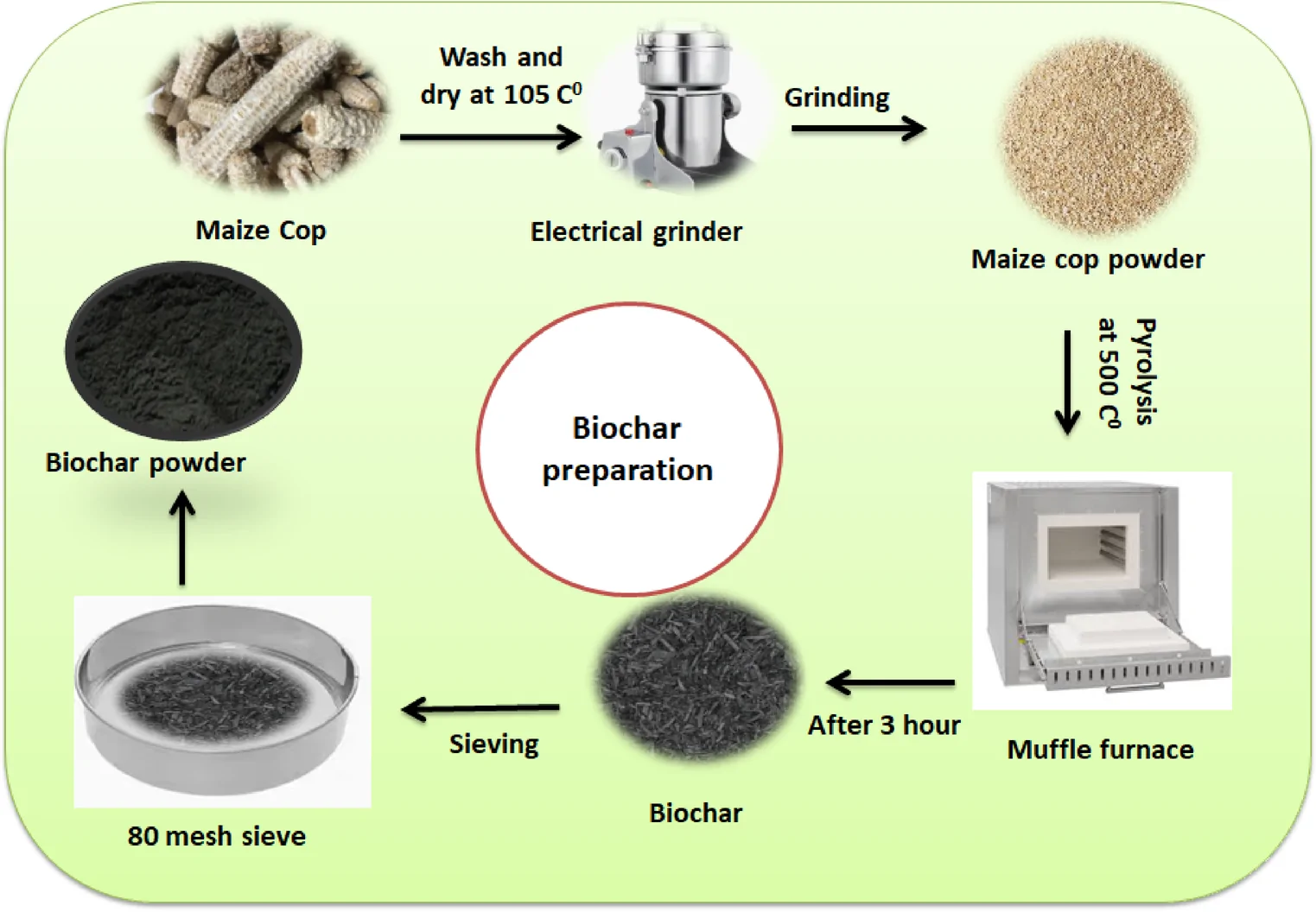
Preparation of biochar with thermal method using muffle furnace at 500 °C.

The feedstock is collected from maize cob waste, obtained from agriculture research centre (ARC) farm located at Giza governorate, the samples were dried at 105 °C with the laboratory oven before embarking on the experimental procedure to extract the moisture. An initial feedstock mass of 200 g was used for each pyrolysis experiment. The pyrolysis experiments were carried out in a box type muffle furnace. The feedstock was placed in a ceramic crucible and subjected to pyrolysis at temperature of 500 °C for 3 h using a heating rate of 10 °C/min under oxygen-limited conditions. After pyrolysis, the biochar sample was left inside the furnace to cool to room temperature then it passed through a 80 mesh-sieve^70^. Approximately 40 g of biochar was obtained after pyrolysis. A graphically illustration of the biochar preparation is shown in Scheme 1.

### Synthesis of ferrites loaded by biochar via the hydrothermal method

**Scheme 2:**
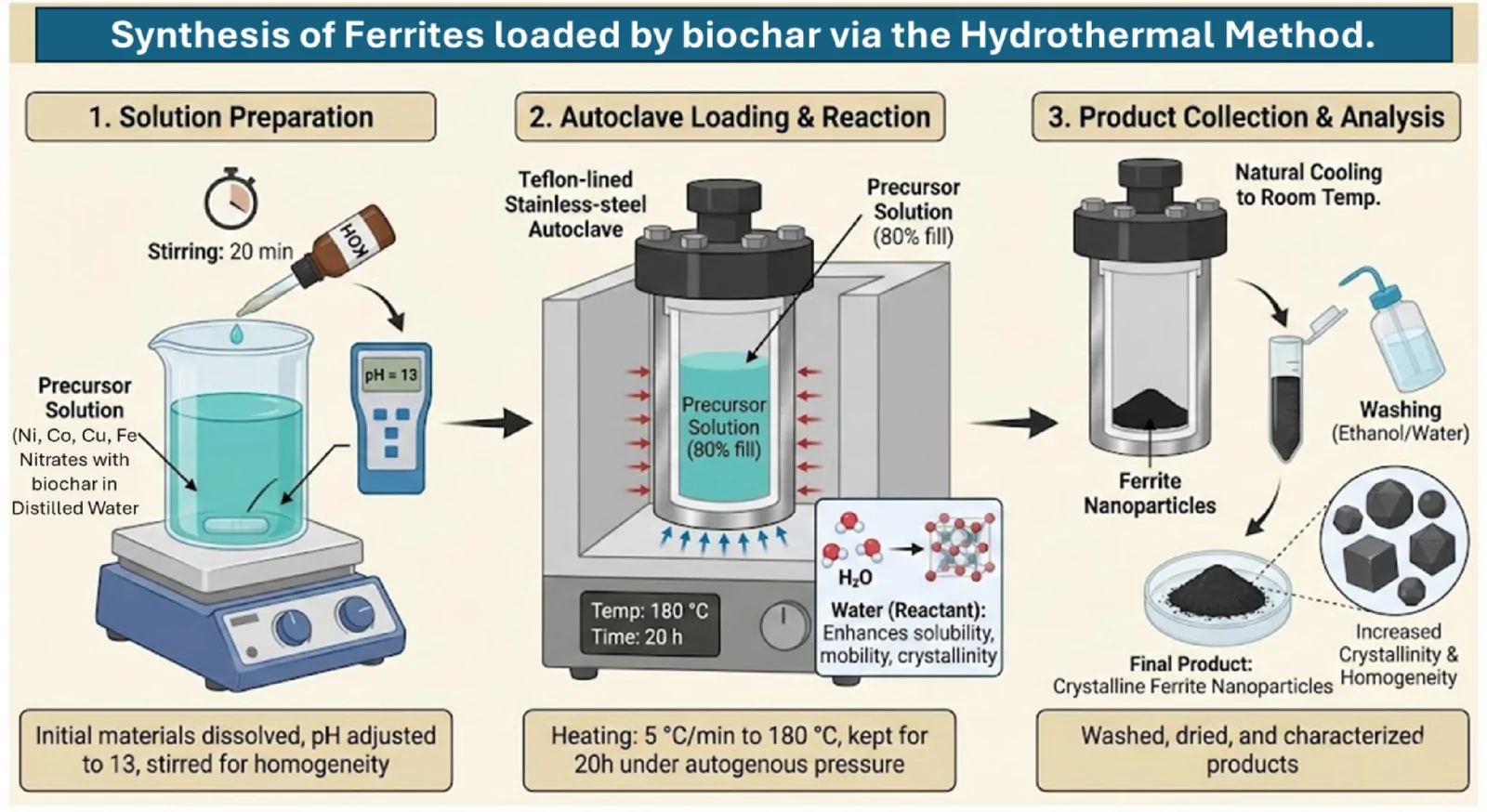
A schematic of preparation method for NCCF/BC composites.

The hydrothermal process is used to prepare the ferrite nanoparticles loaded by biochar, which have the formula Ni₀.₆Co₀.₁Cu₀.₃Fe₂O₄/Biochar (NCCF/BC). Two different biochar loadings were employed: 0.2 g and 0.5 g (BC–0.2, BC–0.5). Typically, high-pressure autoclaves are used to carry out hydrothermal reactions in an aqueous phase. The precursor salts consisted of 8.08 g Fe(NO₃)₃·9H_2_O, 1.745 g Ni(NO₃)₂·6H_2_O, 0.291 g Co(NO_3_)_2_·6H_2_O, and 0.725 g Cu(NO_3_)_2_·3H_2_O, corresponding to the stoichiometric molar ratio of Fe: Ni: Co: Cu = 2:0.6:0.1:0.3 to obtain Ni₀.₆Co₀.₁Cu₀.₃Fe₂O₄. The In the current work, the starting materials were dissolved in sufficient quantities of distilled water, the pH was adjusted to 13 using KOH, and the mixture was stirred for 20 min before being put into a stainless-steel autoclave lined with Teflon. The synthesis took place in an autoclave that was 80% full of the precursor solution and heated at a rate of 5 °C per minute to 180 °C for 20 h, under autogenous pressure, it naturally cooled to room temperature. For comparison, pure Ni₀.₆Co₀.₁Cu₀.₃Fe₂O₄ (NCCF) was synthesized under the same conditions but without the addition of biochar. Scheme 2 shows schematic of preparation method for NCCF/BC composites.

### Structural and surface characterizations

X-ray diffraction (XRD) using (BRUKER, D2 PHASER 2nd gen, Karlsruhe, Germany). applying CuKα radiation at 10 mA and 30 KV. The 2θ range of 5–80° was used to capture the data, with a step size of 0.02° and 0.2 s per step. A sample’s chemical composition was determined using the (Oxford Hitachi XRF X-MET 7000, Buckinghamshire, US). Fourier transformed infrared (FT-IR) spectra in the wavenumber range of 4000–400 cm^− 1^ were measured using FT-IR spectrometers (PerkinElmer 2000, Wellesley, USA). Transmission electron microscope (HRTEM, JEOL TEM-2100, Japan) with a 12,000 X magnification and a 250 kV accelerating voltage. Nitrogen physisorption isotherms, which were essential for determining the surface area and pore size distribution of the synthesized samples, were obtained using the BET method (BELSORP MINI X from MicrotracBEL, Osaka, Japan). Vacuum level: P/P₀ down to ~ 10⁻⁴ for N₂ at 77 K; degassing temperature: 200 °C for 8 h; degas ramp: 10 °C/min; and outgas endpoint criteria: the pressure rise (Δp) ≤ 0.001 (mbar/min). The magnetic properties were characterized using a vibrating sample magnetometer (VSM, Lake Shore 7410, Westerville, OH, USA).

### Adsorption experiment

The adsorption experiment is performed by introducing 0.05 g of the synthesized biochar-based magnetic adsorbent into 25 mL of Pb(II) ions solution at a specified concentration, 30 to 200 ppm, with the pH was previously adjusted. To evaluate the stability of the system, the final pH was measured immediately after reaching equilibrium using a calibrated digital pH meter. The variation between initial and final pH values remained within ± 0.3 units, confirming the controlled hydrogen ion conditions. After that, shaking, at 100 rpm, is conducted for a specific duration, and the filtrate is collected with a nylon syringe filter to measure the residual concentration of the metal ion pollutant in the solution through atomic absorption spectroscopy (NovAA 350, Jena, Germany). All adsorption experiments were performed in triplicate to ensure the results’ reproducibility and accuracy. The relative standard deviation acted as the error parameter for every analysis, with each set of measurements having a value below 5%.

The percentage of removal, R%, and the quantity adsorbed, q_e_, can be calculated using the subsequent equations:

14 $$\:R\%=\:\frac{{C}_{o}-{C}_{e}}{{C}_{o}}\:\times\:100$$

15 $$\:{q}_{e}=\:\frac{{C}_{ads}\times\:V}{m}$$

where, C_o_, C_e_, and C_ads_ represent the concentrations of initial, remaining, and adsorbed metal ions (mg.g^− 1^). V represents the volume of the solution in liters, while m denotes the mass of the adsorbent in grams.

## Conclusion

The ferrite nanoparticles, NCCF, (Ni₀.₆Co₀.₁Cu₀.₃Fe₂O₄) can be successively loaded on different biochar (BC) contents via the hydrothermal technique. Structural and surface characterization confirmed single-phase spinel ferrite and the incorporation of biochar without altering the crystal structure of the ferrite phase. BET surface area and porosity measurements found considerable changes during biochar loading, where NCCF/BC–0.2 exhibited the high surface area, pore volume, and mesoporous structure due to reduced nanoparticle agglomeration and enhanced porosity. In contrast, NCCF/BC–0.5 exhibited a decrease in these properties. This is most likely because the large amount of biochar causes iron oxides to enter and block some of the biochar pores. Magnetic measurements revealed that the incorporation of biochar into the ferrite structure decreases the saturation magnetization while preserving moderate coercivity. Despite this reduction, the resulting composites retain sufficient magnetic response.

Adsorption studies imply that including biochar into the ferrite structure improved adsorption efficiency in acidic conditions. The adsorption follows pseudo-second-order kinetics, suggesting a chemisorption mode through a complexation of surface functional groups. Linear analysis proved that the adsorption data was mostly fitted by the Freundlich model, while nonlinear regression revealed that Freundlich model was suitable for the NCCF and NCCF/BC-0.5 samples, but Langmuir was more suitable for the NCCF/BC-0.2 sample. The maximum adsorption capacities from Langmuir were found to be 388.28 and 217.49 mg g⁻¹. NCCF/BC–0.2 showed the highest adsorption capacity of 388.28 mg g⁻¹, which was significantly higher than those of the bare ferrite and BC. Furthermore, the prepared nanocomposites exhibited good regeneration ability and maintained efficient Pb(II) removal in the presence of competing ions. These findings demonstrate that the prepared magnetic biochar/ferrite nanocomposites, particularly NCCF/BC–0.2, are promising and efficient magnetic biosorbents for wastewater treatment applications.

## Data Availability

The datasets used and/or analysed during the current study available from the corresponding author on reasonable request.
